# Documenting attacks on health workers and facilities in armed conflicts

**DOI:** 10.2471/BLT.15.168328

**Published:** 2016-08-30

**Authors:** Preeti Patel, Fawzia Gibson-Fall, Richard Sullivan, Rachel Irwin

**Affiliations:** a Department of War Studies, King’s College London, Strand, London, WC2R 2LS, England.; b King’s Centre for Global Health, King’s College London, London, England.; c Conflict and Health Research Group, King's College London, London, England.; d Department of Public Health Sciences, Karolinska Institutet, Solna, Sweden.

During armed conflicts, international humanitarian law (which regulates the conduct of parties engaged in war) protects health-care workers and health facilities, the wounded and the sick. In the first half of 2016, however, the international medical charity Médecins Sans Frontières (MSF) reported several attacks on health facilities and workers in Afghanistan, the Central African Republic, South Sudan, the Syrian Arab Republic and Yemen.^1^ These events have attracted media attention to a phenomenon of contemporary armed conflict that has important ramifications for the health, humanitarian, legal and security sectors.^2^ In December 2015, the Stockholm Peace Research Institute and the Conflict and Health Research Group at King’s College London convened a workshop in London on Eliminating violence against health workers: from theory to practice. Participants from MSF, the International Committee of the Red Cross (ICRC), Medical Aid for Palestinians and academic organizations discussed current trends in violence against health workers and attacks on health facilities, presented research findings and highlighted key debates and research gaps in evidence.

Some important lessons can be drawn from ICRC’s Health Care in Danger campaign, MSF’s Medical Care Under Fire campaign, as well as other organizations such as Physicians for Human Rights, which has recently documented mass atrocities in the Syrian Arab Republic as well as the impact of the Syrian conflict on the health sector.^3^^–^^5^ There is a perception of an increase in the number of health workers being killed and facilities being accidentally destroyed (so-called collateral damage) or deliberated targeted during armed conflicts. Comprehensive databases have been set up by independent research organizations to record major incidents of violence against aid workers, such as the Aid Worker Security Database of Humanitarian Outcomes and the Security in Numbers Database from Insecurity Insight.^6^ However, even these do not currently provide health-specific data. The absence of baseline and routine data relating to attacks on health workers and health facilities makes it difficult to identify actual rising trends. Most of the available data sources do not capture violence on local health workers, who seem to bear the brunt of most attacks. Data disaggregated by sex are also lacking.^6^

These gaps in the evidence seem incongruous in an era of increasingly accessible and globalized data. Yet there are many factors that inhibit systematic data collection: poor or non-existent data collection by those in the field (for a variety of reasons ranging from security risks to insufficient research capacity); bias in data collection; insufficient research funding for the topic; and a lack of developed method. Some efforts have been made to monitor and study attacks (both quantitatively and qualitatively), particularly by the ICRC and MSF. However, multidisciplinary, collaborative, long-term retrospective and prospective studies are absent – often for valid reasons. The mandates of some prominent nongovernmental organizations (NGOs) and international organizations may not allow the collection or sharing of such data. If they do allow it, then for perceived reasons of confidentiality and protection, they may not allow these data to be aggregated and analysed, even for meta-analysis by independent organizations. Box 1 summarizes some of the research gaps and needs for better documentation of attacks on health workers and facilities in conflicts.

Box 1 **Key needs for documenting attacks on health workers and health facilities in armed conflicts**

- Analysis of trends of attacks on patients and health-care workers, facilities and transport during armed conflict and other violent incidents.
- Collection of systematic routine data, prospective and retrospective, which are disaggregated by sex.
- Examination of the context of each conflict to understand the dynamics and motives for attacks.
- Disaggregation of data on humanitarian databases to distinguish between types of aid workers, including local and international health-care workers.
- Public availability of anonymized data collected by humanitarian organizations to support a global response on prevention and accountability.
- Assessment of open threats and impact on health facilities and health-care personnel by security staff both before deployment and immediately after conflict.
- Systematic analysis of the immediate and longer-term impact of violence on the providers of health care.

Attacks on health staff may be intentional or unintentional and can take a range of forms: road blockades and checkpoints that delay or block ambulances; attacks against medical personnel, suppliers and patients; direct targeting of hospitals; and armed entry into health facilities.^7^ Political motives for violence and attacks directed at health workers and facilities across several conflict-affected settings include: gaining military advantage; displacing or inducing fear in a population; denying health care to enemies; and seeking health care for the attacker’s own soldiers or affiliates.^7^ Personal disputes between patients and staff, disagreements over treatment and the individual behaviour of both perpetrators and health workers who might resist threats are also among the drivers of violence. The use of violence against health-care facilities to induce fear in the local population has had a resurgence during the current conflicts in the Syrian Arab Republic (which began in 2011) and South Sudan (which escalated in 2013), but this tactic has also been used for example in conflicts in the Democratic Republic of Congo (which began in 1998) and Somalia (from 1988).^3^ Socioeconomic factors, such as poverty and inequality within local populations, as well as inequalities between health workers and local populations, can also be factors in violence against health workers and facilities, because these are viewed as easy targets for looting valuable medicines and equipment. These factors further complicate the task of collecting a robust evidence base.

Overall, one of the most important lessons from research so far is that we must look closely at the context of each attack, to understand its dynamics and motives and to determine responses to attacks and solutions for prevention.^6^ We acknowledge that conducting research on violence towards health workers and facilities during conflict is challenging, particularly in areas where terrorism-related violence is endemic. Humanitarian organizations have a strong operational and long-established local presence, while academic institutions have the capacity to conduct scientifically rigorous, multidisciplinary, quality-assessed and independently corroborated research. Strong research links between these two parties will foster a continuum of knowledge, evidence and practice. This could enable us to develop evidence-based, context-specific guidelines for more effective protection of health workers and facilities during armed conflict. A useful policy lesson comes from the systematic documentation of collateral damage resulting from anti-personnel mines,^8^ whereby the data supported a reduction in the use of such weapons. This strategy could be a model for the health sector to make an effective case to those engaged in conflict (whether national armies or non-state armed groups) to exclude health workers, facilities and patients as deliberate targets. Unless attacks are systematically documented, there will continue to be an important gap in knowledge on the extent and severity of the damage to health-care systems in armed conflict.

We recognize that in many tactical situations, distinguishing between targeted and unintended attacks on health workers and facilities can be problematic. State and multinational armed forces must be encouraged to conduct thorough threat assessments before deployment of forces and during conflicts to prevent and mitigate unintended damage to health workers and facilities. Many armed forces have the technological and intelligence capacities to ensure that, even in fast-changing, unstable situations, tactical awareness of health facilities is possible. Furthermore, after conflicts have ended there should be a requirement for the collection and open distribution of any post-conflict threats of violence against health workers and facilities. This could be done for example through the leadership of the United Nations (UN) Office for the Coordination of Humanitarian Affairs.

There are strong global governance efforts focusing on health care in conflict. These include the 2016 UN Security Council Resolution (2286) on health care in armed conflict, the 2014 UN General Assembly Resolution (A/69/L.35) on global health and foreign policy, focusing on the protection of health workers, and the World Health Assembly’s 2012 Resolution (WHA65.20) calling for leadership from the World Health Organization (WHO) to collect and disseminate data on attacks on health care in complex humanitarian emergencies.^9^ A new system for collecting such data has been developed by WHO and is being tested in the Central African Republic, the Syrian Arab Republic and the West Bank and Gaza Strip. WHO is also field-testing tools to gather data on attacks, and establishing a repository for reports from governments, media and civil society organizations, which was due to be available for use in 2016.^6^^,^^10^ At its 32nd international conference in 2015, the International Red Cross and Red Crescent Movement renewed its commitment to the Geneva Conventions and addressed attacks on health-care personnel and facilities.^11^ These efforts need to be supported with evidence-based research across a variety of conflict-affected contexts to support the important initiatives by humanitarian and human rights NGOs, as well as local emergency responses. Academic institutions have a key part to play in supporting these efforts to improve the evidence base on this pressing humanitarian challenge.^12^
